# Dialysate glucose response phenotypes during peritoneal equilibration test and their association with cardiovascular death: A cohort study

**DOI:** 10.1097/MD.0000000000020447

**Published:** 2020-05-22

**Authors:** Zheng Wang, Dahai Yu, Yamei Cai, Shuang Ma, Bin Zhao, Zhanzheng Zhao, David Simmons

**Affiliations:** a Department of Nephrology, The First Affiliated Hospital, Zhengzhou University, Zhengzhou, China; b Arthritis Research UK Primary Care Centre, Research Institute for Primary Care & Health Sciences, Keele University, Keele, UK; c The Second Division of Internal Medicine, Kejing Community Health Centre, Jiyuan, China; d Western Sydney University, Campbelltown, Sydney, Australia.

**Keywords:** cardiovascular disease, dialysate glucose, mortality, peritoneal dialysis, peritoneal equilibrium test

## Abstract

Different measures of rates of transfer of glucose during the peritoneal equilibrium test (PET), undertaken during peritoneal dialysis (PD) might provide additional information regarding a patient's risk of future cardiovascular mortality. This study aimed to characterize the heterogeneity of dialysate glucose (DG) response phenotypes during the PET and compare the cardiovascular mortality rates associated with the different phenotypes. Our cohort was derived from Henan peritoneal dialysis registry. A total of 3477 patients initiating PD in 2007 to 2014 had the DG measured at 0, 2-hour and 4-hour (D0, D2, and D4 respectively) during the PET for estimation of D2/D0 and D4/D0. Deaths mainly due to CVD within 2 years since the initiation of PD were defined as the outcome. Latent class mixed-effect models were fitted to identify distinct phenotypes of the DG response during the PET. Multivariable unconditional Logistic regression models with adjustment for cardiometabolic risk factors were used to compare the 2-year risk of cardiovascular mortality among patients in the different latent classes. Three distinct DG response phenotypes during the PET were identified. Those with consistently high D2/D0 and D4/D0 ratios had a 1.22 [95% confidence interval: 1.02, 1.35] excess risk of a cardiovascular death within 2 years of commencing PD compared with patients with the lowest D2/D0 ratio and decreased D4/D0 ratio after adjustment for cardiometabolic risk factors. Consistently elevated D2/D0 and D4/D0 ratios during the PET are associated with an increased risk of 2-year cardiovascular mortality independent of other cardiometabolic risk factors. In view of the potential bias due to unmeasured confounders (eg, Family history of cardiovascular diseases, and dietary patterns), this association should be further validated in other external cohorts.

## 1 Introduction

Chronic kidney disease has been recognized as a worldwide health problem.^[1]^ End-stage renal disease (ESRD), in particular, is associated with high premature mortality and a substantial health economic burden.^[2]^ It has been estimated that around 130 million Chinese adults have chronic kidney disease with dialysis needed at some point in their lives.^[3]^ The Chinese medical insurance scheme has now increased its coverage, making dialysis, especially peritoneal dialysis (PD), more affordable among ESRD patients.^[4]^ The leading cause of death among people receiving PD care is cardiovascular disease (CVD).^[5]^

Patients receiving PD routinely undergo a peritoneal equilibration test (PET) to characterize the rate of transfer of solute and water across the peritoneal barrier.^[6]^ The test yields 3 parameters — the 4-hour dialysate to plasma ratio of creatinine (D/P creatinine), the 4- to 0-hour dialysate glucose ratio (D/D0 glucose), and the 4-hour ultrafiltration volume.^[6]^ Although the PET results can facilitate optimizing solute clearances, they are more often used to individualize PD prescriptions to maximize daily peritoneal ultrafiltration.^[7,8]^ Individuals with a faster rate of solute transfer rate experience a higher risk of death, or transfer to hemodialysis, a phenomenon thought to result from volume overload from the challenges of fluid removal during continuous ambulatory PD.^[9]^ Previous research has explored the all-cause death and risk of hospitalization using these 3 measurements at 1 time point: 4-hours after commencing PD.^[10]^ It is not quite clear whether the measurement response phenotypes during the PET are heterogeneous. Such heterogeneity could reflect different underlying pathophysiological mechanisms. These dialysate glucose response phenotypes reflect glucose metabolism status and their association with future risk of cardiovascular death has not been fully explored. Furthermore, investigating whether different dialysate glucose response phenotypes during the PET represent distinct cardiovascular risk profiles may contribute to an understanding of the underlying pathophysiological pathways leading to cardiovascular mortality.

This study now investigates the heterogeneity of dialysate glucose phenotypes based on 2 time points (2-hour and 4-hour) during the PET, and compares the cardiovascular death rate between the identified latent classes. This study hypothesises that there will be patients initializing PD who are at high risk of cardiovascular mortality, who are not captured by the classical 1-time measures of risk.

## 2 Material and methods

### 2.1 Data setting

We have used data from the Henan peritoneal dialysis registry (HPDR) to develop and validate a CVD risk score.^[11]^ Henan is a province in the centre of China with the 2nd largest provincial population. Briefly, as a longitudinal registry dataset, the HPDR is operated under the auspices of the Department of Nephrology, the First Affiliated Hospital of Zhengzhou University and provides an independent audit and analysis of renal care in Henan, China. Information was prospectively collected electronically from all renal units across Henan during the study period. Data arriving at the HPDR are subjected to an algorithm which identifies suspicious values, which are then further verified and corrected where necessary by contacting the renal unit.

### 2.2 Study population

This study was designed as a cohort study derived from HPDR, which includes all adult patients aged 18 years and over who commenced PD between 2007 and 2014 and who had at least 2 years follow-up. Following the standard approach to investigating “real” ESRD patients among all those receiving PD care, and to avoid a reverse causality association between predictors and outcome, patients who died, underwent transplant or whose kidney function recovered within 90 days after initialization of dialysis were excluded (n = 16).

The study was conducted in compliance with the principles of the Declaration of Helsinki and in accordance with Good Clinical Practice guidelines. The protocol was reviewed and approved by the by the Clinical Research Ethics Committee of the First Affiliated Hospital of Zhengzhou University (Reference number: KY-2017-22). All patients providing written informed consent are informed that their anonymized records (with identifiable information removed) may be used for research before study entry.

### 2.3 PET test and dialysate glucose measurements

A standard PET was performed by trained nurses from HPDR; dialysate samples were collected 0, 2-hour, and 4-hour after commencement, and a blood sample was collected at 120 minutes. All blood and dialysate samples were analyzed within 24 hours. Two dialysate glucose ratios: D2/D0 ratio (2-hour dialysate glucose/0-hour dialysate glucose) and D4/D0 ratio (4-hour dialysate glucose/0-hour dialysate glucose) were estimated to quantify the dialysate glucose response phenotypes.

### 2.4 Covariables

All other comorbidity information, laboratory tests, and body measurements were also performed using standard methods at the date of first dialysis.

### 2.5 Statistical analysis

Latent class trajectory analysis was used to identify different dialysate glucose response curve groups (phenotypes) during the PET. The latent class mixed-effects model was specified with linear, quadratic, and cubic time terms to model non-linear change over time.^[12]^ Coefficients were allowed to vary between latent groups. In order to delineate the optimal number of latent groups, we started with 1 group (equivalent to fitting a linear mixed-effects model) and then increased the number of groups one-by one.^[13]^ We repeated this until group sizes remained sufficiently large and the model's Bayesian Information Criteria stabilized.^[9,14]^ After determining the best fitting model, each individual was assigned to the glucose response curve group with the highest membership probability.

Dialysate Glucose response curves were then refitted with standard linear mixed-effects models including group membership and its interaction with all terms within each of the glucose curve groups. We then defined the cardiometabolic characteristics (age, sex, body mass index [BMI], lipid profile, phosphate, estimated glomerular filtration rate [eGFR], systolic blood pressure [SBP] and diastolic blood pressure, respectively) of study participants stratified by glucose response curve group.

Missing information was as follows: BMI (10.51%), phosphate (10.23%), albumin (9.92%), total protein (TP) (12.75%), total cholesterol (TC) (14.53%), low density lipoprotein (14.58%), high density lipoprotein (14.52%), triglyceride (14.52%), fasting glucose (FG) (15.26%), SBP (2.26%), and DBP (2.23%). We used multiple imputation to replace missing values by using a chained equation approach based on all candidate predictors. Fifteen imputed datasets were created for missing variables that were then combined across all datasets by using Rubin rule to obtain estimations.

Multivariable unconditional logistic regression models, with adjustment for 2 sets of variables, were used to compare the risk of cardiovascular mortality between latent classes. Model 1 was adjusted for age and gender. Model 2 was additionally adjusted for other variables presented in Table 1.

**Table 1:**
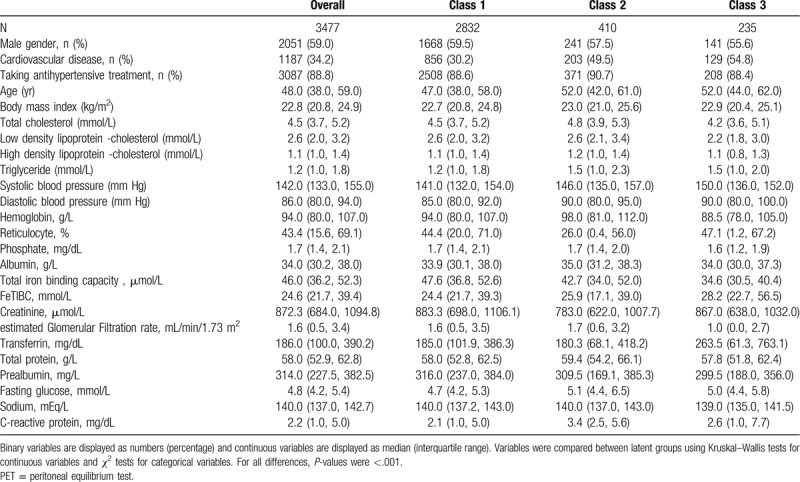
Demographic and cardiometabolic risk factor characteristics by dialysate glucose response phenotypes during the PET.

All analyses were performed using STATA (STATA/SE 14.0 StataCorp, College Station, TX). All *P*-values were calculated using 2-tailed tests and a *P*-value < .05 was taken to indicate statistical significance.

## 3 Results

**Figure 1:**
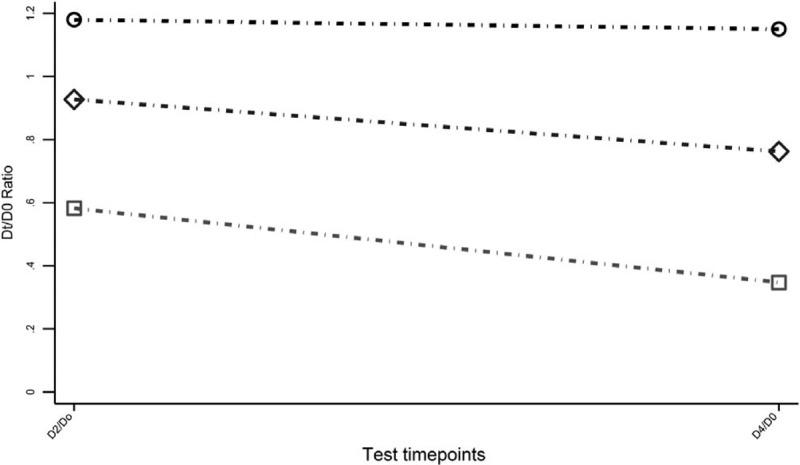
Phenotypes of dialysate glucose ratios in the peritoneal equilibration test. Grey square dash-dot line, Latent class 1 (n = 2832, 81.5%); grey diamond dash-dot line, Latent class 2 (n = 410, 11.8%); black circle dash-dot line, Latent class 3 (n = 235, 6.7%). D0 = dialysate glucose at 0 h, D2 = dialysate glucose at 2 h, D4 = dialysate glucose at 4 h.

Three latent classes were identified, each including 6.7%, 11.8%, and 81.5% of the cohort (Fig. 1). Average posterior group membership probabilities were high, ranging between 0.81 and 0.91, indicating good discrimination between the groups. The D2/D0 ratio varied between groups from 0.58 to 1.17, and the D4/Do ratio ranged from 0.41 to 1.15, indicating considerable heterogeneity.

Class 1 demonstrated the lowest D2/D0 and D4/Do ratios with a dramatic decrease in glucose over the 2 time points. Class 3 had the highest D2/D0 and D4/D0 ratios with stable estimations over the 2 time points. Class 2 demonstrated a glucose decrease response phenotype midway between Class 1 and Class 3 (Fig. 1).

Characteristics of study participants stratified by dialysate glucose response curves are shown in Table 1. Class 1, characterized by the lowest median age (47 years), the highest proportion of males (59.5%), the lowest proportion of having previously diagnosed CVD, the lowest median BMI, blood pressure, triglyceride, FG and C-reactive protein, and the highest median creatinine and prealbumin concentrations.

Patients in Class 2 had the highest proportion of prescribed antihypertensive medication, the highest median TC, high density lipoprotein cholesterol, hemoglobin (HGB), albumin, eGFR, TP, FG, and C-reactive protein, and lowest median level of serum creatinine.

Class 3 had the lowest proportion of males, the highest proportion of having previously diagnosed CVD, the lowest median level of TC, low density lipoprotein cholesterol, HGB, phosphorus, Total iron binding capacity, eGFR, TP, prealbumin, and sodium, and the highest median level of blood pressure, reticulocyte, Fe-total iron binding capacity, and transferrin.

Absolute numbers and mortality rates with adjusted odd ratios for 2-year cardiovascular mortality are provided in Table 2. The 2-year mortality rates were 10.3 (95% confidence interval: 9.2, 11.5) %, 10.0 (7.3, 13.3) %, and 15.7 (11.3, 21.0) % for Class 1, Class 2, and Class 3, respectively. In the most adjusted model, patients in Class 3 had a 22% elevated risk (odds ratio 1.22 [95 confidence interval: 1.09, 1.35]) of CVD death compared with those in Class 1. Those in Class 2 had no excess CVD mortality risk.

**Table 2:**
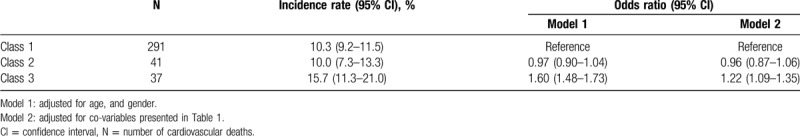
Crude incidence rates and adjusted odds ratios (95% confidence interval) for cardiovascular mortality by latent class (phenotypes).

## 4 Discussion

This study identified heterogeneity in dialysate glucose response to the PET test by a novel latent class trajectory analysis approach. This study revealed 3 different phenotypes and was able to associate them with the major long-term outcome, CVD death. Class 3, when compared with Class 1 and had a 22% increased CVD mortality, accompanied by a worse cardiometabolic risk profile. This high-risk group had a stable estimation of dialysate glucose over the PET and remaining at the highest dialysate glucose/D0 estimations. Others have found that D4/D0, is a marker for all-cause mortality and hospitalization and this is in line with our study.^[10]^

The higher CVD mortality in patients in Class 3 might be explained by their higher proportion of existing CVD, which has been reported in other studies.^[15,16]^ A further possible explanation lies with their higher SBP: prior observation studies have reported a strong association between high SBP and CVD mortality.^[15,17]^ Those in Class 3 also had worse kidney function, as measured by eGFR, another risk factor for future CVD deaths, as previously shown.^[18]^ Risk factors generally reflecting worse health status, like HGB and TP were also lower in Class 3, which could also have contributed to the increased risk of CVD mortality as observed in another study.^[19–21]^

In previous studies, the 1 time-point D4/D0 ratio has been used to predict the future risk of long-term adverse outcomes.^[22]^ However, such measurements have limitations, as individual patients with different dialysate glucose response trajectories can still have similar values for some or several calculated curve features. Therefore, instead of studying predefined curve characteristics, we considered heterogeneity of change using a data-driven approach. Latent class trajectory analysis allows the investigation of change over time, while taking measurement error into account. Compared with conventional approaches assessing only mean growth curves, the latent class method is suitable for revealing heterogeneous phenotypes, which may give a more complete picture of associations.

It is important to bear in mind that the aim of this study was not to derive the best prediction model or to determine optimal cut-off values for the examined outcomes, but to find associations that might not have been revealed using conventional methods. We observed that different dialysate glucose response phenotypes during PET are associated with CVD death, which persist after adjustment for (eg, cannot be fully explained by differences in) the cardiometabolic risk profile. This suggests that dialysate glucose measurements at different time points should be considered simultaneously to obtain a more detailed picture of the individual risk of future CVD death. Although the dialysate glucose response pattern was measured and recorded by trained nurses from HPDR to ensure relatively high validity, involvement of only a single recruitment site could have led to information bias. Therefore, replication studies in external datasets are warranted. Another limitation of this study that warrants further investigation is the difference in CVD mortality rates between classes. Numbers in categories 2 and 3 were low and hence power was insufficient to examine in detail the different causes for the excess CVD mortality rates in Class 3 compared with Class 1. Moreover, our results might be influenced by confounding by unmeasured risk factors, for example, those due to under-recording of pre-existing comorbidities and their duration. The duration of reduced renal function would be a particularly important measure prone to under-estimation. Third, we lacked data on lifestyle factors, including smoking, drinking, and physical activity, although reliability of such measures is always a potential concern. Fourth, we did not adjust for PD patients’ socioeconomic status which could influence patient treatment/management and general health status. Finally, the proportion of clinical measurement data that was missing was relatively high, requiring multiple imputation, which suggesting that further replication in the external dataset are warranted.

## 5 Conclusions

In conclusion, a consistently similar high ratio of D2/D0 and D4/D0 was associated with a high risk of future CVD mortality. Latent class analysis seems to be a promising method to reveal otherwise unidentified subgroups that do not fit into the risk category defined by D4/Do alone. However, in view of the potential bias due to unmeasured confounders (eg, Family history of CVDs, and dietary patterns) and the involvement of a single recruitment site, this association should be further validated in other external cohorts.

## Acknowledgments

The authors thank the First Affiliated Hospital of Zhengzhou University approved this study. The authors thank the Henan Peritoneal Dialysis Registry (HPDR) to provide the data for this study.

## Author contributions

**Conceptualization:** Dahai Yu, Yamei Cai, Zhanzheng Zhao, David Simmons.

**Data curation:** Dahai Yu, Yamei Cai, Bin Zhao, Zhanzheng Zhao.

**Formal analysis:** Dahai Yu, Yamei Cai, Bin Zhao, David Simmons.

**Funding acquisition:** Zhanzheng Zhao.

**Investigation:** Zheng Wang, Dahai Yu, Shuang Ma, Bin Zhao, Zhanzheng Zhao.

**Methodology:** Dahai Yu, Yamei Cai, Shuang Ma, Bin Zhao, Zhanzheng Zhao, David Simmons.

**Project administration:** Zheng Wang, Shuang Ma, Bin Zhao, Zhanzheng Zhao, David Simmons.

**Resources:** Zheng Wang, Shuang Ma, Zhanzheng Zhao.

**Software:** Yamei Cai, David Simmons.

**Supervision:** Zheng Wang, Bin Zhao, David Simmons.

**Validation:** Dahai Yu, Yamei Cai, Bin Zhao.

**Visualization:** Dahai Yu, Yamei Cai, Zhanzheng Zhao, David Simmons.

**Writing – original draft:** Zheng Wang, Dahai Yu, Yamei Cai, David Simmons.

**Writing – review and editing:** Zheng Wang, Dahai Yu, Yamei Cai, Shuang Ma, Bin Zhao, Zhanzheng Zhao, David Simmons.
